# RNA-Seq Analysis of Microglia Reveals Time-Dependent Activation of Specific Genetic Programs following Spinal Cord Injury

**DOI:** 10.3389/fnmol.2017.00090

**Published:** 2017-04-03

**Authors:** Harun N. Noristani, Yannick N. Gerber, Jean-Charles Sabourin, Marine Le Corre, Nicolas Lonjon, Nadine Mestre-Frances, Hélène E. Hirbec, Florence E. Perrin

**Affiliations:** ^1^MMDN, University of Montpellier; EPHE, Institut National de la Santé et de la Recherche Médicale U1198Montpellier, France; ^2^Institut National de la Santé et de la Recherche Médicale U1051Montpellier, France; ^3^“Integrative Biology of Neurodegeneration”, IKERBASQUE Basque Foundation for Science and Neuroscience Department, University of the Basque CountryBilbao, Spain; ^4^Department of Neurosurgery, Gui de Chauliac HospitalMontpellier, France; ^5^Institute for Functional Genomics, CNRS UMR5203, Institut National de la Santé et de la Recherche Médicale U1191Montpellier, France

**Keywords:** spinal cord injuries, microglia, RNA sequencing, DNA damage, BRCA1, non-human primate

## Abstract

Neurons have inherent competence to regrow following injury, although not spontaneously. Spinal cord injury (SCI) induces a pronounced neuroinflammation driven by resident microglia and infiltrating peripheral macrophages. Microglia are the first reactive glial population after SCI and participate in recruitment of monocyte-derived macrophages to the lesion site. Both positive and negative influence of microglia and macrophages on axonal regeneration had been reported after SCI, raising the issue whether their response depends on time post-lesion or different lesion severity. We analyzed molecular alterations in microglia at several time-points after different SCI severities using RNA-sequencing. We demonstrate that activation of microglia is time-dependent post-injury but is independent of lesion severity. Early transcriptomic response of microglia after SCI involves proliferation and neuroprotection, which is then switched to neuroinflammation at later stages. Moreover, SCI induces an autologous microglial expression of astrocytic markers with over 6% of microglia expressing glial fibrillary acidic protein and vimentin from as early as 72 h post-lesion and up to 6 weeks after injury. We also identified the potential involvement of DNA damage and in particular tumor suppressor gene *breast cancer susceptibility gene 1* (*Brca1*) in microglia after SCI. Finally, we established that BRCA1 protein is specifically expressed in non-human primate spinal microglia and is upregulated after SCI. Our data provide the first transcriptomic analysis of microglia at multiple stages after different SCI severities. Injury-induced microglia expression of astrocytic markers at RNA and protein levels demonstrates novel insights into microglia plasticity. Finally, increased microglia expression of BRCA1 in rodents and non-human primate model of SCI, suggests the involvement of oncogenic proteins after CNS lesion.

## Introduction

Spinal cord injury (SCI) is a devastating condition with clinical symptoms that depend on both the anatomical level of the injury and the severity of the lesion. SCI triggers microglia activation, peripheral macrophages and neutrophil infiltration that together govern neuroinflammation after central nervous system (CNS) injury (David and Kroner, 2011). Microglia, the main immune cells of the CNS, are the first responsive glial cells after SCI (Tian et al., 2007) and participate in recruitment of monocyte-derived macrophages to the lesion site (David and Kroner, 2011). Microglia and monocytes have myelomonocytic origin that may explain their common expression of surface receptors and signaling molecules (Schmitz et al., 2009; Kettenmann et al., 2011).

Although activation of microglia/macrophages promotes inflammation and tissue damage after SCI, these cells may also have neuroprotective actions (David and Kroner, 2011). In particular, microglia play major roles in the phagocytosis of cellular debris (Perrin et al., 2005) and express neurotrophic factors (Lambertsen et al., 2009) that account for their beneficial effects after SCI (Mukaino et al., 2010). Similarly, other studies have reported both positive (Shechter et al., 2009) and negative (Popovich et al., 1999) influence of infiltrating monocyte-derived macrophages on functional recovery after SCI. Together, these studies highlight positive and negative influence of CNS resident microglia and peripheral infiltrating macrophages after SCI. However, whether these dual roles are due to differences in time post-injury, lesion severity or both remains unclear and the precise molecular mechanisms that control the positive and negative roles of activated microglia post-injury are poorly understood.

When studying microglia it is important to take into account other closely related cells such as macrophages, monocytes and dendritic cells. This is particularly important in CNS trauma, in which destruction of the blood-CNS barrier induces infiltration of inflammatory cells. Microglia prominently express a Gαi-coupled seven-transmembrane receptor, the chemokine receptor CX3CR1. The generation of CX3CR1^eGFP^ transgenic mice, which express enhanced Green Fluorescent Protein (eGFP) downstream of the *Cx3cr1* promoter, provided a powerful research tool to potentially isolate microglia using techniques such as flow cytometry (Jung et al., 2000; Wolf et al., 2013). Importantly, CX3CR1 is also expressed in circulating monocytes and resident macrophages (Jung et al., 2000; Gautier et al., 2012). However, recent studies have revealed additional markers, including Ly6C, which can be used, at least partially, to distinguish microglia from these additional cell types (Chiu et al., 2013; Butovsky et al., 2014; Gosselin et al., 2014; Bennett et al., 2016).

Transcriptomic analyses of microglia at multiple time-points after different lesion severities provide a powerful approach to reveal their exact molecular involvements in SCI pathophysiology. Recent findings, including our own, using gene expression profiling in microglia have unveiled novel insights into their contribution in multiple neuropathologies (Olah et al., 2012; Parakalan et al., 2012; Beutner et al., 2013; Chiu et al., 2013; Hickman et al., 2013; Noristani et al., 2015b). To this end, using cell-specific microarrays in CD11b^+^ microglia, we recently identified DNA damage pathway and in particular the tumor suppressor gene *breast cancer susceptibility gene 1* (*Brca1*) upregulation in an animal model of amyotrophic lateral sclerosis (ALS) (Noristani et al., 2015b). Furthermore, we identified BRCA1 as a novel human microglial marker and demonstrated its specific over-expression in ALS microglia within the spinal cord (Noristani et al., 2015b).

Although rodents are routinely used to study SCI pathophysiology, there are significant differences in neuroanatomical organization and neurophysiological features between rodents and primates that is a major obstacle in translation to clinics (Courtine et al., 2007; Friedli et al., 2015). In a recent study, using contusive SCI in common marmosets (*Callithrix jacchus*), analysis of changes in gene expression established that the inflammatory response is prolonged and the glial scar formation is delayed in non-human primates as compared to rodents (Nishimura et al., 2014). Indeed, expression levels of cytokines and immune cell markers that were maximal at 1 week after injury decreased by 2 weeks post-SCI concomitant with the onset of glial scar formation (Nishimura et al., 2014). Thus, in order to improve translational research, experimental findings in rodents need to be compared with those seen in non-human primate models of SCI.

In the current study, we used CX3CR1^+/eGFP^ transgenic mice and flow cytometry to isolate microglia at several stages after different lesion severities and carried out RNA sequencing (RNA-Seq) on the isolated cells. We analyzed the transcriptomic signature of microglia after hemisection (HS) and full transection (FT) at 3, 7, and 14 days following lesion relative to that of non-injured (NI) controls. We chose these two SCI models to study microglia responses in environments with different regeneration capacity; namely FT that shows no regeneration and lateral HS because limited spontaneous regeneration does occur between 3 days and 2 weeks post-lesion due to the presence of spared tissue (Lee and Lee, 2013). Our results revealed distinct transcriptional deregulation in microglia that is primarily driven by time post-lesion, irrespective of lesion severity. Strikingly, we also found that following SCI over 6% of microglia express classical astrocytic markers including glial fibrillary acidic protein (GFAP) and vimentin. Microglia expression of astrocytic markers was evident as early as 72 h, peaking by 1 week post-lesion and continued up to 6 weeks after both HS and FT SCI. We also identified the potential involvement of DNA damage pathway and BRCA1 in microglia following SCI. Finally, we extended these results to spinal cord injured non-human primate and confirmed an alteration of BRCA1 expression specifically in microglia after SCI. These data represent the first transcriptomic analysis of microglia at multiple time-points after different spinal cord lesion severities and thus provide novel insights into the response profile of these cells following SCI.

## Materials and methods

### Ethical approval

Experimental procedures followed the European legislative, administrative and statutory measures for animal experimentation (2010/63/EU). The study was approved by the “Direction des Services Vétérinaires de l'Hérault,” the regional ethic committee for animal experimentation, France and the “Ministère de l'éducation nationale, l'enseignement supérieur et de la recherche” (authorization number 34118 for mice and CEEA-LR-12142 for non-human primates).

### Experimental procedures

Transgenic mice expressing eGFP in CNS resident microglia and peripheral monocytes (CX3CR1^+/GFP^) were obtained from Dr. Dan Littman, Howard Hughes Medical Institute, Skirball Institute, NYU Medical Centre, New York, USA and maintained on a C57BL/6 background (The Jackson Laboratory, Bar Harbor, ME, USA) (Jung et al., 2000). Mice were housed in controlled conditions (hygrometry, temperature and 12 h light/dark cycle). Five adult male gray mouse lemurs (*Microcebus murinus*, 2 years of age) were also used in the current study. *Microcebus murinus* were all born and bred in the animal facility (CECEMA, University of Montpellier, France). *Microcebus murinus* were housed together in cages (2 × 1 × 1 m, 3 lemurs per cage) until surgery, and then separately (60 × 60 × 50 cm) for 1 week following injury before they were returned to their original cages. All cages were equipped with wooden nests. *Microcebus murinus* were kept at standard temperature (24–26°C) and relative humidity (55%) and fed with fresh fruits and a daily-made mixture of cereals, milk and eggs. Water and food were given *ad libitum*. Twenty-four hours after surgery *Microcebus murinus* were given flour worm to increase their protein intake.

### Spinal cord injury

Adult heterozygote CX3CR1^+/GFP^ mice (12 weeks of age) were anesthetized by inhalation of 1.5% isoflurane gas; laminectomy was performed and lateral (HS) or (FT) were carried out under microscope using a micro knife [10315-12, Fine Science Tools (FST)], as previously described (Noristani et al., 2015a, 2016). Lesions were done at thoracic 9 (T9) level to obtain complete paraplegia (FT) or hemi paraplegia (HS) while preserving complete respiratory function.

For SCI in *Microcebus murinus*, atropine (0.4–0.6 mg/kg) was administered subcutaneously 15 min prior to the surgery to inhibit salivary and bronchial secretions as well as vagal stimulation. Anesthesia on *Microcebus murinus* was induced with 3–4% of isoflurane and maintained with 1–2% isoflurane and 1 L/min oxygen flow rate throughout the surgery. The skin and muscles overlying the lumbar segment were cut along the back midline and a laminectomy was carried out. Lateral (HS) of the spinal cord was done at lumbar level 1 (L1) under microscope using a micro knife. Muscles and skin were sutured and animals were left to recover on a temperature-controlled pad.

#### Postoperative cares

In mice, bladders were emptied manually twice daily until recovery of full sphincter control (in the case of HS) or throughout the 6 weeks of the study (in case of FT group). Bodyweights were measured before surgery and daily throughout the 6 weeks period after injury.

Lemurs were observed twice daily. Bodyweights were measured daily until stabilization, then once a week. Buprenorphine (0.01 mg/kg/day) and enterofloxacine (5 mg/kg/day) were administered via intramuscular and subcutaneous routes, respectively, for up to 1 week after the injury. Animals were kept for 3 months after lesion.

### Flow cytometry

Flow cytometry was used to obtain microglia populations at multiple stages after both HS and FT in CX3CR1^+/GFP^ mice. We used a 1 cm-segment centered on the lesion site to isolate eGFP^+^ microglia. For non-injured (NI) control mice, an equivalent 1 cm-thoracic segment was used. Male CX3CR1^+/GFP^ mice were anesthetized using tribromoethanol (500 mg/kg) and intracardially perfused with 0.1 M RNAse-free phosphate base saline (PBS, Invitrogen, Carlsbad, USA). Spinal cords were thoroughly dissected in a cocktail of 750 μl PBS, 100 μl of 13 mg/ml trypsin, 100 μl of 7 mg/ml hyaluronidase, 50 μl of 4 mg/ml kinurenic acid (all from Sigma Aldrich, Saint Louis, USA) and 20 μl of 10 mg/ml DNAse I (Roche, Rotkreuz, Switzerland) for 30 min at 37°C. Cell suspension was sieved using 40 μm sieve (BD Biosciences, Franklin Lakes, USA), re-suspended in 0.9 M sucrose and centrifuged for additional 20 min at 750 × g to remove the myelin sheath. Cell pellet was re-suspended in 500 μl of 7AAD (1 μl/ml, Sigma Aldrich, Saint Louis, USA). Microglia were sorted using FACSAria (BD Biosciences, Franklin Lakes, USA), equipped with a 488 nm Laser Sapphire 488–20. Size threshold was used to eliminate cellular debris. Sorted microglia were then centrifuged for 5 min at 700 g and re-suspended in 250 μl of RLT lysis buffer (Qiagen, Maryland, USA) containing 1% beta-mercaptoethanol.

In a parallel experiment designed to better define the selected cell population, we used 2 non-injured mice, 8 mice at 72 h after HS and 7 mice at 1 and 2 weeks following HS. Pelleted cells collected after sucrose gradients (as described above) were re-suspended in PBS, first incubated for 10 min at 4°C with Fc-receptor block CD16/32 antibody (1:100; BD Biosciences, Franklin Lakes, USA) and then 30 min at 4°C with mouse anti-CD11b-PE (1:200; BD Pharmingen, San Diego, USA), CD45-APC (1:200; BD Pharmingen, San Diego, USA), LY6C-PE-Cy7 (1:200; BD Biosciences, Franklin Lakes, USA). FACS analyses were performed using the FACSAria (BD Biosciences, Franklin Lakes, USA). All flow cytometry data were analyzed using the FlowJo Software (Ashland, Oregon, USA).

### RNA sequencing

Total RNA was isolated using the RNeasy Mini Kit, (Qiagen, Maryland, USA) with DNAse treatment. The quality of starting RNA and amplified cRNA were tested (Agilent 2100 bioanalyzer, RNA 6000 Pico LabChip, Palo Alto, USA) and used only when the RNA integrity number (RIN) was >7, as previously described (Kiewe et al., 2009). Total RNA was isolated from mice at 72 h, 1 and 2 weeks post-injury (*n* = 29 for HS and *n* = 48 for FT) and from non-injured (NI) controls (*n* = 38). RNA-Seq was performed on the polyadenylated fraction of RNA using HiSeq 2500 (Ilumina, San Diego, USA). Three biological replicates were used for all time-points including NI control. 10 ng of total RNA was used for each replicate library. FastQC was used to assess sequencing quality control. FASTX-Toolkit was used to remove the reads containing adapter sequences. Filtered reads were mapped with TopHat v2.0.9 software to use the UCSC mm10 release reference on new junctions and known annotations. RSeQC v2.3.3, PicardTools v1.92 and SamTools v.1.18 software were used to verify biological quality control and summarization. HTSeq v.0.5.4p3 was used to prepare the count data. R/Bioconductor edgeR software v.3.3.6 was used to carry out data normalization and differential expression analyses. Genes that achieved 10 counts in at least two replicates were kept, whereas very low expressed genes were filtered out. The filtered data were normalized by the library size and differentially expressed genes were estimated using negative binomial general model statistics. To identify differentially expressed transcripts, we defined a criterion of a 2-fold and greater difference plus a significant (*p* < 0.05) false discovery rate (FDR). Statistics: *t*-test with un-equal variance. The R/Bioconductor MFuzz package v.2.18.0 was used for fuzzy clustering of the differentially expressed genes over time in both HS and FT compared to NI controls. Pathway analysis was performed using MetaCore (Thomson Reuters), as described previously (Noristani et al., 2015b, 2016). Accession number for RNA-Seq data: Gene Expression Omnibus (GE0) GSE96055.

#### Primary antibodies for immunological staining

Primary antibodies used included rabbit anti GFAP (1:1000; Z 0334; Dako, Glostrup, Denmark), mouse anti GFAP (1:1000; G3893; Sigma Aldrich, Saint Louis, USA), chicken anti GFP (1:1000; Ab13970; Abcam; Cambridge, UK), mouse anti vimentin (1:500; V2258-2ML; Sigma Aldrich, Saint Louis, USA), rabbit anti IBA1 (1:1000; 019-19741; Wako Pure Chemical Industries, Osaka, Japan), rabbit anti BRCA1 (1:100, Santa Cruz Biotechnology, Dallas, USA), rabbit anti KI67 (1:500; NCL-Ki67p; Leica, Novocastra, Wetzlar, Germany) and rabbit anti S100 beta (1:1000, (DAKO, Denmark).

### Immunohistochemistry

Hemisected and non-injured control female mice as well as hemisected *Microcebus murinus* were anesthetized via intraperitoneal injection of tribromoethanol (500 mg/kg) or ketamine, respectively and then intracardially perfused using cold 0.1 M PBS followed by 4% paraformaldehyde (PFA, Sigma Aldrich, Saint Louis, USA). Spinal cords were carefully removed and post-fixed for additional 2 h in 4% PFA at room temperature, cryoprotected in 30% sucrose, included in Tissue Tek (Sakura, Alphen aan den Rijn, The Netherlands), frozen and kept at −20°C until processing. Frozen spinal cords were cut either longitudinally (20 μm, mice only) or transversally (14 μm, mice and *Microcebus murinus*) and collected on Superfrost Plus© slides.

For fluorescence immunohistochemistry, slides were washed in 0.1 M PBS and incubated for 20 min in 20 mM lysine (pH 7.2). To block non-specific labeling, sections were incubated for 1 h in 1% bovine serum albumin (BSA, Sigma Aldrich, Saint Louis, USA) and 0.1% Triton X-100 (Fisher Scientific, Illkirch, France). Sections were then incubated in primary antibody for 48 h at 4°C. Slides were then washed in 0.1 M PBS and incubated with secondary antibodies conjugated to Alexa 488, 594, or 633 (1:1000, Vector Laboratories, Burlingame, USA and Millipore Bioscience Research Re-agents, Massachusetts, USA) and with the nuclear stain 4′,6-diamidino-2-phenylindole dihydro-chloride (DAPI, 2 ng/ml, Invitrogen, Massachusetts, USA). Sections were coverslipped using fluorescence mounting medium (DAKO, Denmark).

For peroxidase immunohistochemistry, sections were treated for 30 min in 20 mM lysine followed by incubation in 1% hydrogen peroxide (H_2_O_2_) for 30 min. Sections were blocked for 1 h with 0.1 M PBS containing 1% BSA and 0.1% Triton X-100 and then incubated for 48 h at 4°C with primary antibody. Slides were rinsed with 0.1 M PBS for 30 min and incubated in 1:1000 dilutions of the corresponding peroxidase-coupled secondary antibody (Jackson Immunoresearch, UK) for 1 h at room temperature. Slides were then rinsed with 0.1 M PBS. The peroxidase reaction product was visualized by incubating in a solution containing 0.022% of 3,3′diaminobenzidine and 0.003% H_2_O_2_ for 30 min. The reaction was stopped by rinsing the sections in 0.1 M PBS for 15 min. Slides were then dehydrated in ascending ethanol concentration and finally xylene. Coverslips were applied using Entellan (Merck KGaA, Germany). Morphometric fluorescence photographs were obtained using a fluorescent microscope (Zeiss, Axio Imager M1, Oberkochen, Germany), a laser scanning inverted (Leica SP5, Mannheim, Germany) and confocal microscopy (Zeiss 5 Live Duo, Oberkochen, Germany) to assess co-expression of two proteins. 225 × 225 μm^2^ images were taken adjacent to the lesion center for each animal and the co-localization coefficient was measured between the two channels. In total we assessed co-localization within a 6750 × 6750 μm^2^ surface area adjacent to the lesion site. Laser intensity and detector sensitivity settings were kept constant for all acquisition of all images in a given experiment. 5–7 animals were used per time-point. Co-localization analysis was performed using the Carl Zeiss LSM-710-NLO software. Morphometric bright field photographs were obtained and analyzed using NanoZoomer RS slide scanner (NanoZoomer Digital Pathology System and NDP view software, Hamamatsu, Japan).

### Relative optical density

To determine SCI-induced changes in BRCA1 expression in *Microcebus murinus*, the mean optical density (OD) was measured at different distances 3 months after spinal cord (HS) using ImageJ (National Institutes of Health, USA), as described previously (Noristani et al., 2010). OD is a sensitive and reliable method to measure expression level of a given signal and to detect changes caused by different experimental conditions. Following peroxidase immunostaining, spinal cord sections were scanned using Nanozoomer RS slide scanner that uses constant light intensity and exposure time to obtain peroxidase-labeled photographs (NanoZoomer Digital Pathology System and NDP view software, Hamamatsu, Japan). Digital images were exported with RGB color profiles, using identical exposure settings for all sections. To avoid potential variation in staining intensity between different slides or animals, we carried out BRCA1 immunostaining of all animals in parallel. BRCA1 intensity was analyzed in at least fifty sections throughout the lesion segment of the spinal cord with 210 μm intervals. Non-specific background was determined for individual section and subtracted from the general signal. Optical density measures included the gray matter, the white matter and the dorsal funiculus of the spinal cord. By clearly distinguishing lesion epicenter (section with highest damaged area) from un-injured tissue, we were able to determine differences in BRCA1 intensity along the entire segment of the spinal cord both above and below the injury site.

### Statistical analysis

Paired *t*-tests were used to compare differences in BRCA1 intensity at similar distance between above and below the lesion site in *Microcebus murinus*. Significance was defined as *p* ≤ 0.05. The data were analyzed using GraphPad Prism 5.0 (GraphPad Software, Inc, CA, USA). All data are shown as the mean ± standard error of the mean (SEM).

## Results

### Time-dependent microglia/macrophages responses after SCI

To study microglia responses after SCI, we used chemokine (C-X3-C Motif) Receptor 1 (CX3CR1^+/eGFP^) transgenic mice that selectively express enhanced green fluorescent protein (eGFP) in CNS resident microglia and peripheral monocytes (Jung et al., 2000). Dual immunohistochemistry in uninjured mice, using ionized calcium binding adaptor molecule 1 (IBA1) confirmed specific microglial eGFP expression in spinal cord of CX3CR1^+/eGFP^ mice (Figures S1A–C). Moreover, immunostaining using S100 beta (Figures S1D–F), GFAP (**Figures 3C–E**) and vimentin (**Figures 3L–N**), established that in non-injured CX3CR1^+/eGFP^ mice eGFP is not expressed in astrocytes. Following injury, no co-localization of eGFP and S100 beta was observed (Figures S1G–I) ruling out potential expression of CX3CR1 by reactive astrocytes.

To study neuroinflammation at early and chronic stages after injury, we used flow cytometry to isolate eGFP^high^ expressing cells at 72 h, 1 and 2 weeks post-lesion in both HS and FT. No eGFP expression was observed in wild type control littermate (Figure S2A). Sorted eGFP^high^ expressing cells (Figures S2A–D, red dots) were further analyzed for RNAseq. In a parallel experiment, to better characterize the selected eGFP^high^ cell population, we used a combination of CD45, CD11b and LY6C antibodies in both non-injured and pathological conditions (all time points after HS). Ramified microglia express CD11b^+^/CD45^low^ cells whereas fully activated microglia and infiltrating macrophages are CD11b^+^/CD45^high^ cells (David and Kroner, 2011). At 72 h after SCI it was possible to distinguish 2 sub-populations of eGFP^+^ cells based on CD45 expression level (Figure S2F). These sub-population most likely correspond to ramified microglia (CD45^low^) and activated macrophages (CD45^high^). In contrast, at 1 and 2 weeks post-injury this discrimination was not possible (Figures S2G,H). Further combination of CD45 and LY6C showed that at all time points, eGFP^high^ cells were LY6C^−^, strongly suggesting that the majority of selected cells were microglia (Figures S2I–U). Even if we cannot exclude contamination with infiltrating macrophages, we will further refer to the selected population as microglia.

**Figure 1:**
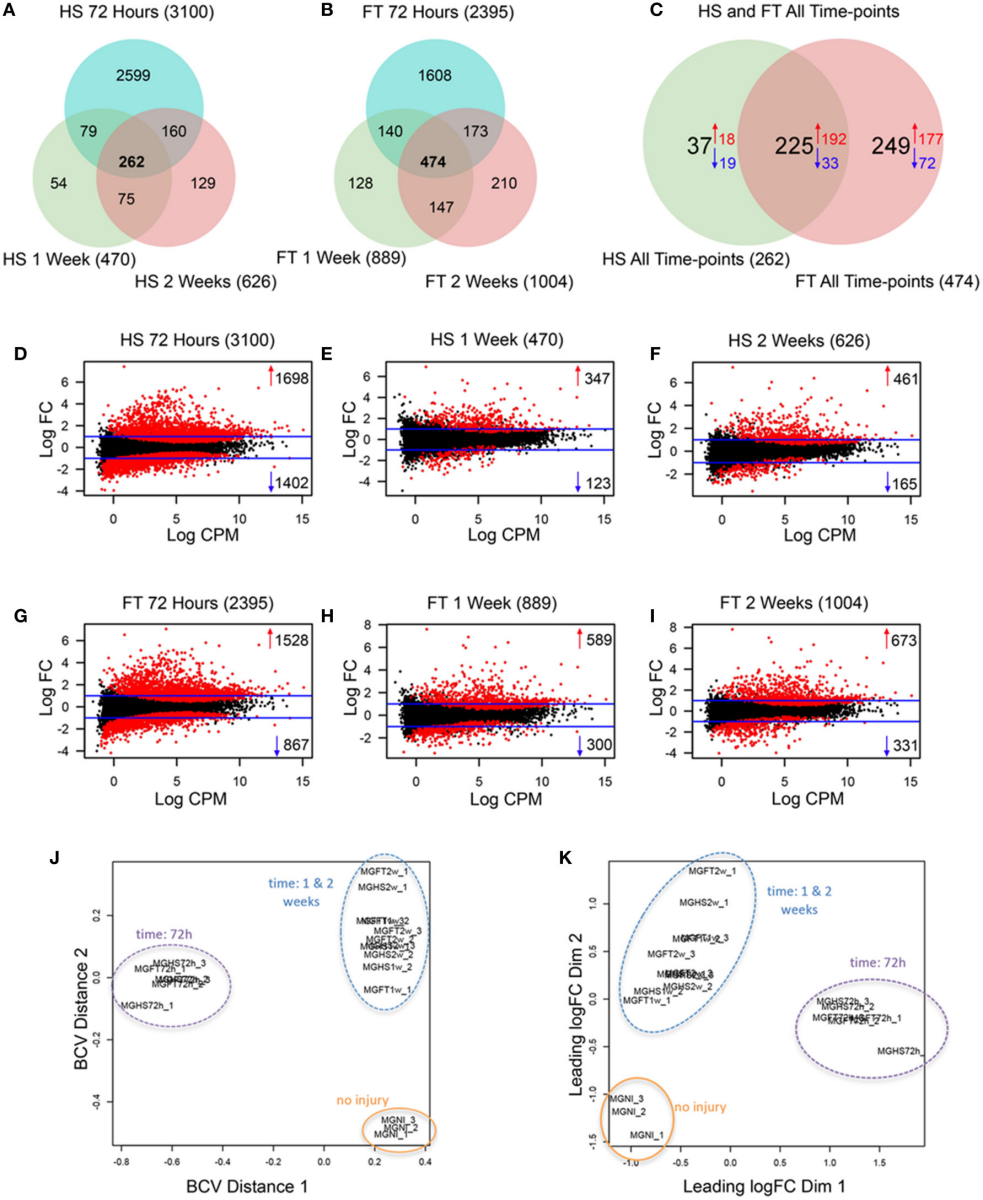
**Longitudinal analyses of de-regulated genes in microglia after 2 severities of SCI**. **(A–C)** Venn diagrams displaying the number of deregulated genes in microglia isolated by flow cytometry at the indicated stages after HS and FT. Relationship between average expression and log 2 of the fold-change in microglia at different stages after HS **(D–F)** and FT **(G–I)** injuries. Horizontal blue lines indicate the cut off criterion used to define deferentially expressed genes with a FC >2 (logFC comprised between–1 and 1) and a significant false discovery rate (FDR) values (*p* < 0.05). Red points indicate significantly deregulated genes following injury. Multidimensional scaling plots displaying distances, in terms of biological coefficient of variation **(J)** and fold change **(K)** in microglia at different stages after HS and FT injuries relative to NI control.

SCI induced a similar increase in microglia number after HS and FT SCI compared to NI control (Figures S2B–D) and RNA from isolated microglia was of high quality (RIN > 7, data not shown). We then carried out RNA-Seq analyses to determine transcriptomic changes in microglia at multiple time-points after the two SCI severities (Tables S1, S3). We found an increased expression of microglia and monocyte markers (including *Arg1, Ccr2*, and *Emr1*) (Mildner et al., 2007; Gautier et al., 2012; Zhang et al., 2014; Greenhalgh et al., 2016) that were independent of lesion severity (Figure S3A, Table S3). In all samples we detected only very low expression and no lesion-induced change in neuronal (*Tubb3, Isl1, Syt1* and *Gabra1*), astrocytic (*Aldh1l1, Tnc and Aqp4*) or oligodendrocyte (*Sox10, Mag, Mog, Mobp, Cx47*, and *Cldn11*) transcripts, further confirming the purity of our isolation procedure (Table S3). To identify differentially expressed (DE) transcripts, we defined a criterion of a 2-fold and greater difference plus a significant (*p* < 0.05) false discovery rate (FDR). We identified 3100, 470, and 626 DE genes in HS as well as 2395, 889, and 1004 DE genes in FT group at 72 h, 1 and 2 weeks post-lesion compared to NI control, respectively (Figures 1A,B). 225 genes were deregulated in both HS and FT groups at all time-points, whilst only 37 and 249 genes were specifically deregulated in HS and FT relative to NI control, respectively (Figure 1C). Overall, microglia displayed a greater number of upregulated genes compared to down regulated genes (Figures 1D–I). In both injury severities, a higher number of DE genes was observed at 72 h compared to 1 and 2 weeks post-lesion. Comparisons between the two injury severities revealed a greater number of DE genes after HS compared to FT injury at 72 h (Figures 1D,G). However, at 1 and 2 weeks post-lesion FT displayed a greater number of DE genes compared to the HS group (Figures 1E,F,H,I). To determine the relationship between gene expression profiles at different time-points post-lesion, we generated a multidimensional scaling plot (Szulzewsky et al., 2016). Samples from NI mice clustered together and samples from injured animals clustered according to time after injury but not lesion severity (Figures 1J,K).

To provide a more in-depth analysis, we directly compared the total number of differentially expressed genes between the two injury severities at all 3 time-points (comparison number 7, 8, and 9 Figure S4A). No differences in gene expression were found upon direct comparison between HS and FT groups at 72 h whilst only 8 dysregulated and 1 up-regulated transcripts were found at 1 and 2 weeks, respectively (Figures S4B–E).

Taken together, these findings demonstrate that (1) SCI induces a higher number of upregulated than down regulated transcripts in microglia, (2) at 72 h after HS injury microglia express a higher number of DE genes than FT, (3) the number of DE genes in microglia at 1 and 2 weeks after lesion are higher after FT than in HS and (4) microglia responses after injury are time- but not severity-dependent.

### Microglia responses after SCI involve early anti-inflammatory response and proliferation

**Figure 2:**
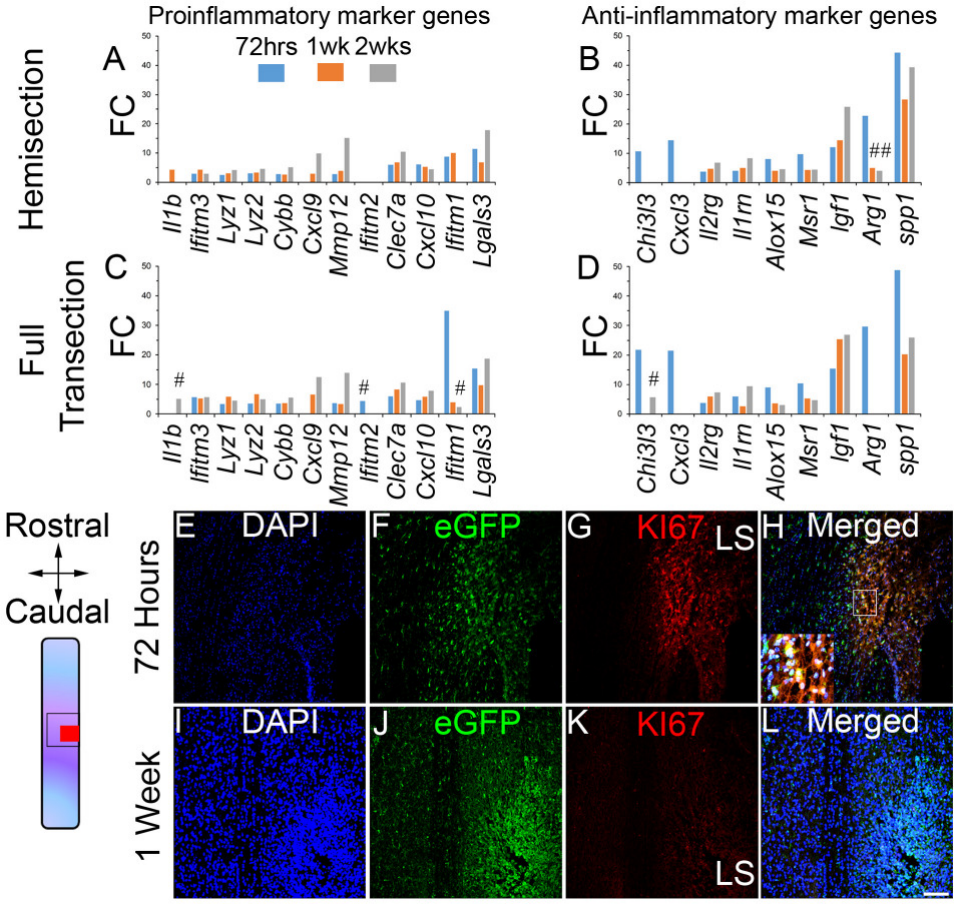
**Acute inflammatory response and proliferation of microglia after SCI**. Bar graphs displaying alterations in pro-inflammatory and anti-inflammatory marker genes in microglia after HS **(A,B)** and FT **(C,D)**. Values are actual fold change. # represents transcripts with different expression between the two lesion severities at the same time-points after injury. Schematic drawing of longitudinal spinal cord sections from HS groups illustrating the lesion site (red square) and reference frames for displayed field of views. Confocal micrographs indicating proliferation of microglia at 72 h after SCI **(E–H)**. Proliferation of microglia ceased by 1 week after injury **(I–L)**. Scale bars **(E–L)**, 100 μm. LS, lesion site. **(E–H)** HS 72 h and **(I–L)** HS 1 week. FC, fold change.

Activation of microglia has been described as neurotoxic (also known as M1) and neuroprotective (also known as M2) states (Hickman et al., 2013; Kroner et al., 2014), although, more recent findings suggest a multidimensional model of activation (Xue et al., 2014; Ginhoux et al., 2016; Ransohoff, 2016). We identified significantly increased expression of 12 pro-inflammatory and 9 anti-inflammatory transcripts after injury (Figures 2A–D). Anti-inflammatory transcript upregulation was more evident at 72 h post-lesion (9/9 transcripts, following both HS and FT, Figures 2B,D) compared to pro-inflammatory markers (9/12 transcripts after HS and 10/12 transcripts after FT, Figures 2A,C). Moreover, the level of over expression was overall higher for anti-inflammatory (Figures 2B–D) compared to proinfammatory markers (Figures 2A–C). Comparison of DE genes between the two injury models revealed only minor differences. In particular, the FT group showed slight upregulation of pro-inflammatory, and down regulation of anti-inflammatory, transcripts (Figures 2C,D), compared to HS. It is, however, important to note that altered abundance of this limited number of transcripts should not be used to definitively infer microglial pro vs. anti-inflammatory roles in the context of SCI.

To provide insight into the potential roles of microglia at different stages after injury, we carried out pathway analysis of DE genes in the two experimental models at the three time-points following lesion (Noristani et al., 2015b, 2016). At 72 h post-lesion, microglial responses predominantly involved proliferation as indicated by up-regulation of numerous genes involved in cell cycle processes (Table S2). We confirmed proliferation of microglia at 72 h post-lesion using KI67, which is exclusively expressed by actively dividing cells (Figures 2E–H). By 1 week after lesion proliferation had ceased (Figures 2I–L) and microglial responses included inflammation, defense response, cytoskeleton and extracellular matrix remodeling (Table S2). Pathway analysis comparison between the two lesion severities also showed no differences between FT and HS groups, further confirming that microglial responses after injury is mainly driven by time post-lesion.

Altogether, these data suggest that (1) acute microglial responses to injury involve anti-inflammatory signaling, (2) proliferation of microglia principally occurs within 72 h post-lesion, (3) by 1 and 2 weeks following CNS lesion microglia regulate multiple aspects of immune response and (4) responses of microglia after injury differ only slightly between the two lesion severities.

### SCI induces expression of astrocytic markers in microglia

**Figure 3:**
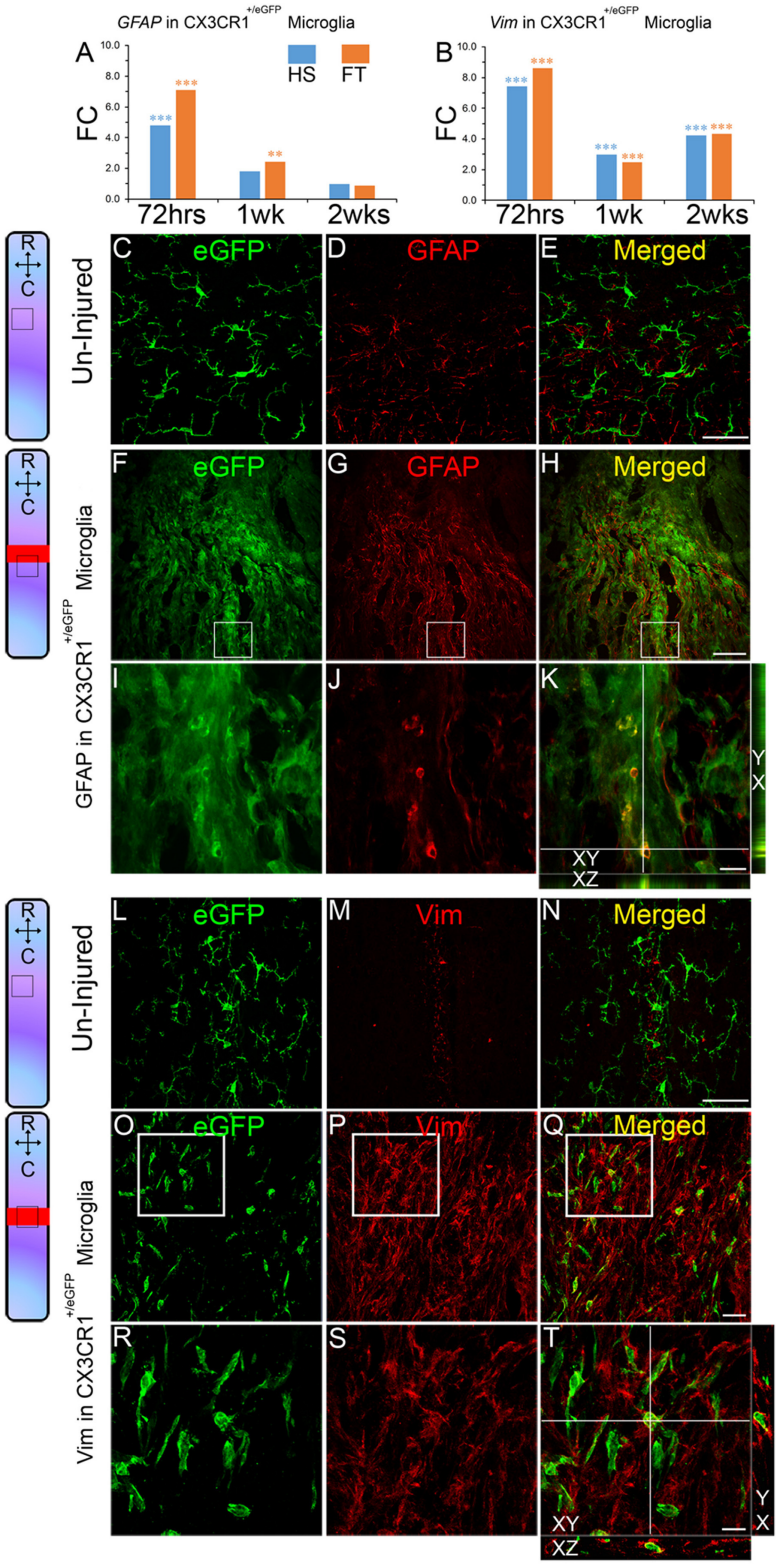
**Astroglial marker expression by microglia after SCI**. Bar graphs indicating up-regulation of *Gfap* and *Vim* transcripts expressions in microglia after SCI **(A,B)**. Values are actual fold change (^**^p < 0.01; ^***^p < 0.001 by *t*-test). Schematic drawing of longitudinal spinal cord sections from non-injured control and after FT illustrating the lesion site (red rectangle) and reference frames for the displayed fields of view. Confocal micrographs of longitudinally cut non-injured spinal cord section showing no eGFP/GFAP **(C–E)** or eGFP/vim **(L–N)** co-expression. Following SCI eGFP/GFAP **(F–K)** and eGFP/vim **(O–T)** protein co-expressions were evident in a sub-population of microglia in CX3CR1^+/eGFP^ mice. Small insets in **K,T** are orthogonal projections of confocal z-stacks, which show eGFP co-localization with GFAP and vim, respectively (confocal z-stack with XY, XZ, and YX views). Scale bars **(C–E,L–N)**, 50 μm; **(F–H)**, 100 μm; **(O–Q)**, 25 μm; **(I–K, R–T)**, 10 μm. LS, lesion site. **(C–E,L–N)** non-injured and **(F–K,O–T)** FT 2 weeks.

Interestingly, our pathway analysis of microglial transcriptional changes also suggested the involvement of a “neural stem cell lineage” pathway at 1 week after FT (Table S2) due to increased expression of astrocyte-specific marker genes including *vimentin* (*Vim*) and *glial fibrillary acidic protein* (*Gfap*) (Figure S5). *Gfap* transcript levels were significantly increased at 72 h in both HS and FT and in FT only 1 week after injury, while *Vim* transcript levels were significantly increased at all time points after both HS and FT injuries (Figures 3A,B). Using immunohistochemistry, we confirmed protein expression of both GFAP and VIM in CX3CR1^+/eGFP^ microglia after SCI (Figures 3F–K,O–T). In contrast, GFAP/eGFP or VIM/eGFP co-expression was not observed in non-injured CX3CR1^+/eGFP^ spinal cords (Figures 3C–E,L–N). In the injured spinal cord, GFAP-expressing microglia were primarily located within 500 μm distance adjacent to the lesion site whereas VIM-positive microglia were mainly found within the lesion epicenter (Figures 3F–H,O–Q). Morphologically, GFAP and VIM proteins were predominantly located in somata and primary processes (Figures 3I–K,R–S). SCI-induced astrocytic marker expression by microglia was also supported by pronounced upregulation of *Serpin Family A Member 3* transcripts (*Serpina3n*), a marker of reactive astrocytes (Zamanian et al., 2012) (Figures S3B,C). *Serpina3n* transcript expression increased more than 20-fold at 72 h post-lesion and 30-folds after HS and FT SCI, respectively (Figures S3B,C). In addition, at 72 h after FT we also observed a 2.4-fold increased expression of the pan-astrocytic marker *Aldh1l1* (Cahoy et al., 2008). No changes were observed in other astrocyte-specific transcripts (Table S3).

**Figure 4:**
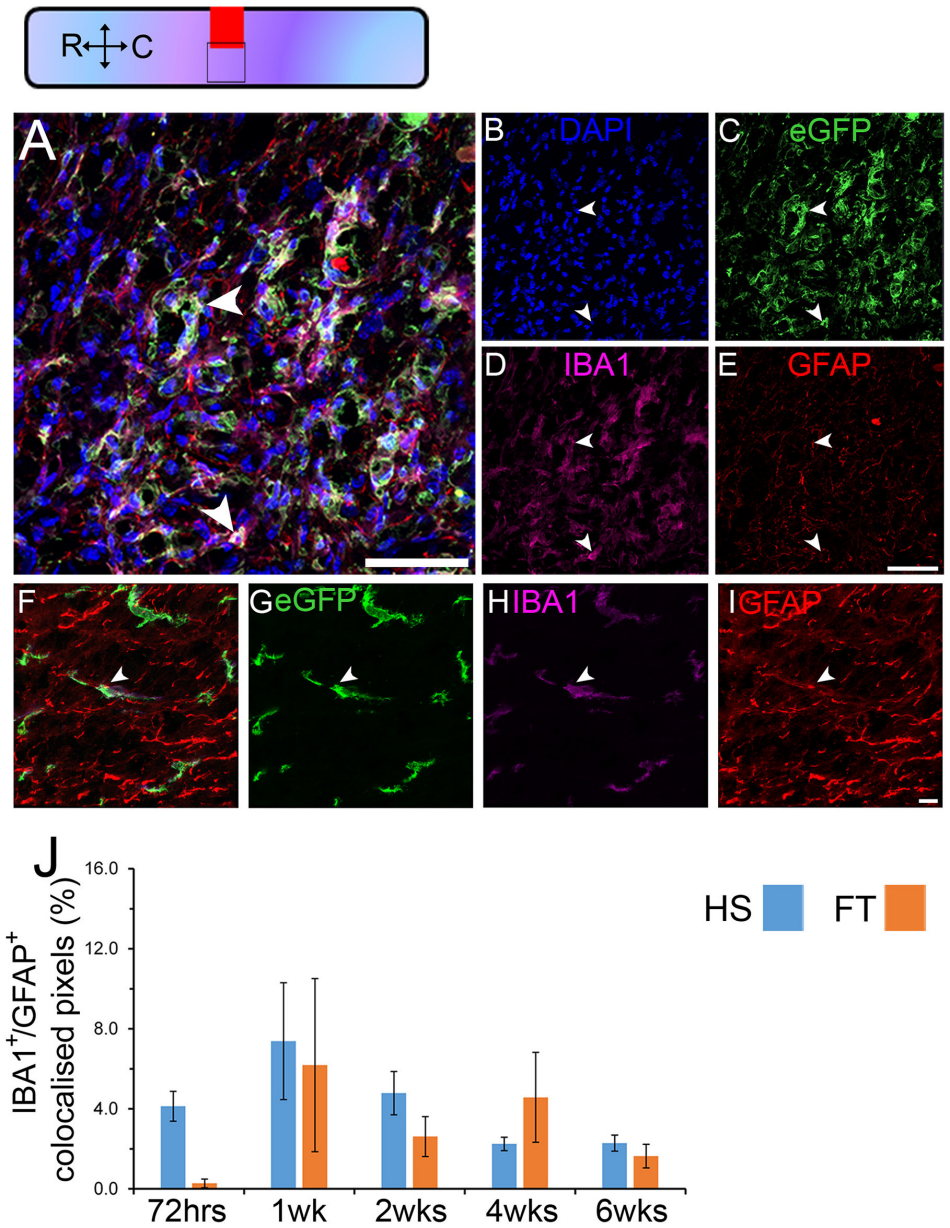
**Astrocytic marker expression in microglia continues up to 6 weeks after different lesion severities**. Schematic of longitudinal spinal cord section after HS SCI illustrating the lesion site (red square) and reference frames for the displayed fields of view. Confocal micrographs showing DAPI **(B)**, eGFP **(C)**, IBA1 **(D)** and GFAP **(E)** in CX3CR1^+/eGFP^. Micrographs were taken from regions adjacent to the lesion site (overlap of red and black squares in the schematic view). Higher magnification images, acquired from the same spinal cord, further from the lesion (black square without overlap with the lesion in the schematic view) confirmed the presence of IBA1/GFAP co-expressing cells **(F–I)**. Quantitative analysis of IBA1/GFAP co-expressing cells (arrowheads in **A,F**) in the 2 lesion severities at multiple stages after SCI **(J)**. Scale bars **(A–E)**, 50 μm; **(F–I)**, 10 μm. Bars represent mean ± SEM. 225 × 225 μm^2^ images were taken adjacent to the lesion center for each given animal and the colocalization coefficient were measured between the two channels. In total we assessed colocalization within 6750 × 6750 μm^2^ surface area adjacent to the lesion site in 5–7 animals at each given time-point. Colocalization analysis was performed using the Carl Zeiss LSM-710-NLO software. **(A–E)** HS 4 weeks, **(F–I)** HS 1 week.

We next examined SCI-induced expression of astrocytic markers in microglia of adult spinal cord at multiple stages after different lesion severity (Figure 4, Table S3). Time-course analyses in CX3CR1^+/eGFP^ mice showed that astrocytic marker expression in microglia starts as early as 72 h after injury and peaks at 1 week post-lesion reaching 7.4 and 6.2% in HS and FT groups, respectively (Figure 4J). By 6 weeks post-lesion only 2.3 and 1.6% of microglia continued to express GFAP in the HS and FT groups, respectively (Figure 4J).

These data demonstrate that expression of astrocytic markers in microglia is evident at 72 h after injury, peaks at 1 week post-lesion and decreases to lower levels up to 6 weeks (the longest time-point investigated in this study) after both HS and FT SCI.

### Breast cancer 1 (Brca1) pathway involvement in microglia after SCI

**Figure 5:**
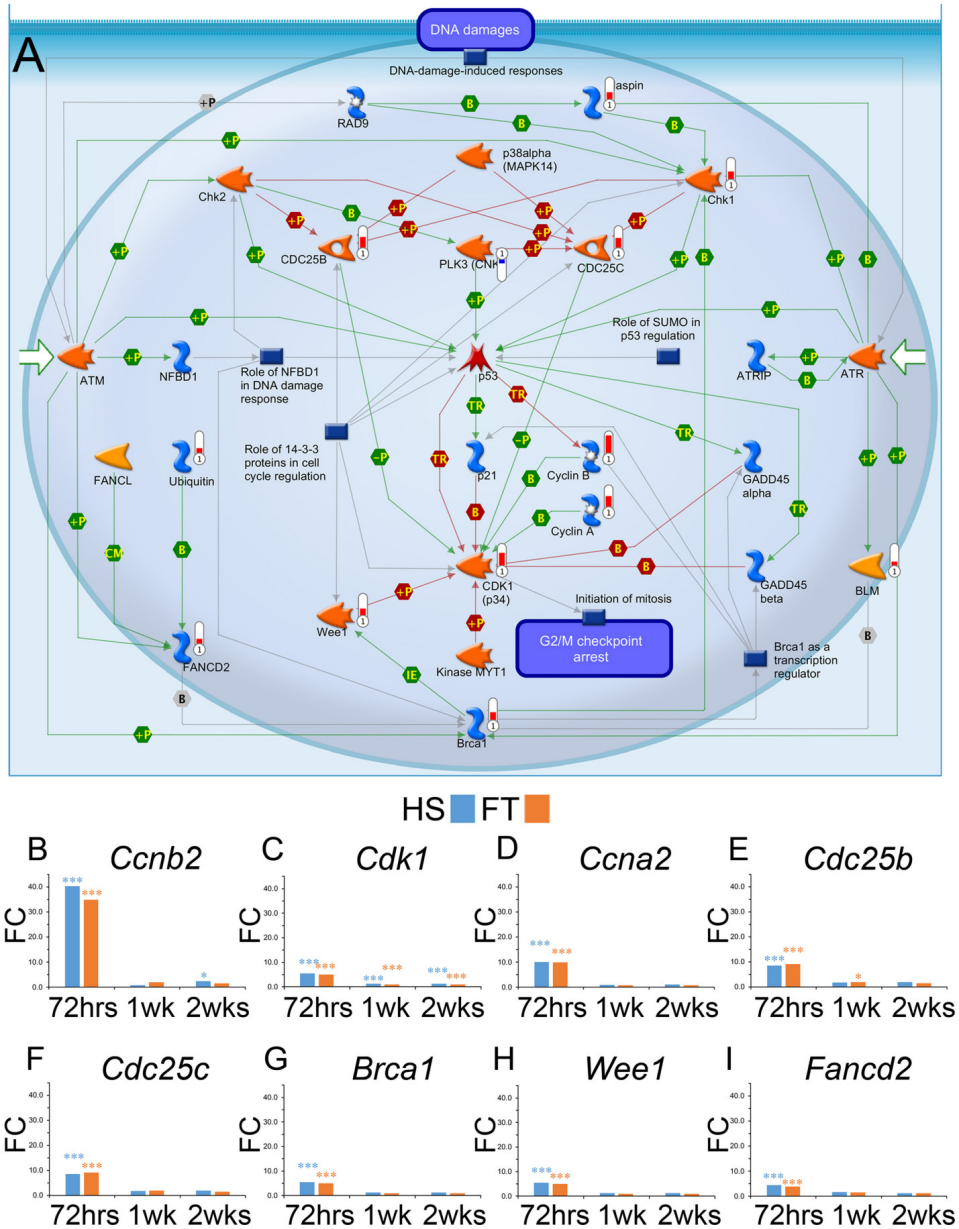
**Induction of DNA damage pathway genes in microglia after SCI**. Gene ontology pathway map analysis displaying the induction of DNA damage pathway in microglia after SCI **(A)**. Thermometers indicate deregulated transcripts (red, up-regulated; blue, down-regulated). Interactions between objects, green (positive or activation); red (negative or inhibition); gray (unspecified); B: Binding (physical interaction between molecules); 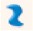
Binding protein, 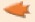
Kinase, 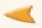
Generic enzyme. Bar graphs displaying over-expression of transcripts involved in DNA damage pathway at different time-points after HS and FT injuries **(B–I)**. Values are actual fold change, t-test between HS and FT at a given time point (^*^p < 0.05; ^***^p < 0.001).

Pathway analyses also identified DNA damage in microglia at 72 h after HS SCI (Table S2; Figure 5A) with over 65% (17/26) of deregulated transcripts from this pathway (Table S2). A more in-depth time-course analysis of DE transcripts involved in the DNA damage pathway revealed a consistent deregulation that peaked 72 h after both HS and FT SCI (Figures 5B–I). Concomitant dysregulation of these genes pointed toward a potential involvement of *Brca1* as a key transcription regulator (Figure 5A).

**Figure 6:**
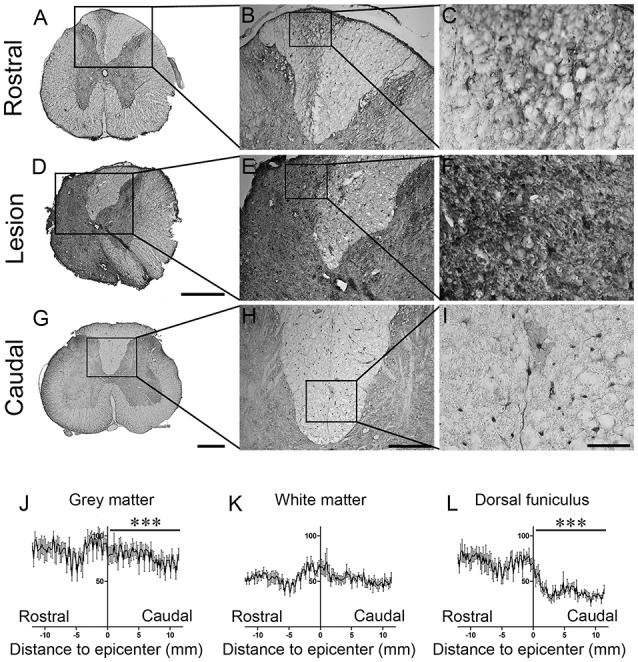
**Increased BRCA1 reactivity 3 months after SCI in *Microcebus murinus***. Bright field micrographs displaying BRCA1-positive microglia rostral **(A–C)**, within **(D–F)** and caudal **(G–I)** to the lesion site 3 months after spinal cord hemisection in *Microcebus murinus*. Note that BRCA1-positive microglia displayed a characteristic ramified morphology with long/thin processes caudal to the lesion sites **(I)**, whilst activated/amoeboid microglia had enlarged cell bodies with short/thick processes that were mainly evident within the dorsal funiculus rostral to the lesion site **(C)** and adjacent to the lesion epicentre **(I)**. Line graphs displaying quantitative assessment of BRCA1-positive microglia reactivity within the gray matter **(J)**, the white matter **(K)** and the dorsal funiculus **(L)** of the spinal cords at different distances to the lesion epicentre. Note the increase in BRCA1 reactivity within the gray matter and the dorsal funiculus **(l)** of the spinal cord rostral to the lesion site as compared to caudal regions after lesion. ^***^P < 0.001 paired *t*-test compared to similar distance rostral to the lesion site. Scale bar **(A,D,G)**, 500 μm; **(B,E,H)**, 200 μm; **(C,F,I)**, 50 μm.

To determine whether SCI-induced BRCA1 over-expression in microglia is observed across species, we carried out immunohistochemistry in spinal cord injured *Microcebus murinus*, a non-human primate (Figures 6A–I). Morphologically, BRCA1-positive microglia in *Microcebus murinus* could be subdivided into two groups: (a) ramified/resting with small cell bodies and thin-to-medium-size processes that were randomly distributed (Figure 6I), and (b) activated/amoeboid with enlarged cell bodies and short/thick processes (Figures 6C,F). Activated/amoeboid microglia were evident not only within the lesion site but also in the dorsal funiculus (Figures 6A–F), whereas only ramified microglia were observed caudal to the lesion (Figures 6G–I). To determine which cell population express BRCA1, we carried out immunohistochemistry using adjacent sections (Figures S6A–I), because autoflorescence in primate spinal cord prevented us from performing dual-label fluorescent immunostaining. IBA1 immunostaining demonstrated identical pattern and cell morphology to that of BRCA1, strongly suggesting microglial expression of BRCA1 in *Microcebus murinus*.

Finally, quantitative analyses in *Microcebus murinus* demonstrated a pronounced increase in BRCA1 expression adjacent to the lesion site compared to rostral and caudal segments of the spinal cords both in the gray and the white matters (Figures 6J,K). In addition, increased BRCA1 expression was also found specifically within the dorsal funiculus rostral to the lesion site (Figures 6A–C,L).

These data demonstrate that BRCA1 overexpression is induced by SCI in microglia of both rodents and non-human primates.

## Discussion

**Figure 7:**
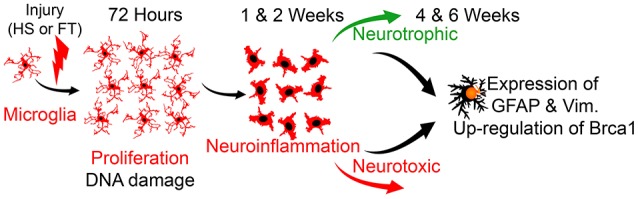
**Schematic cartoon illustrating microglial response after SCI**. Microglia undergo rapid proliferation post-injury, followed by a predominant role in neuroinflammation, irrespective of lesion severity. Early after SCI, microglia also express markers associated with DNA damage pathway and in particular *Brca1*. Microglia displayed a dual phenotype with increased expression of both pro- and anti-inflammatory factors after both HS and FT SCI. Concurrently, a small percentage of microglia co-express astrocyte-specific markers at acute and chronic stages after SCI.

Findings of the current study indicate that microglia undergo specific transcriptomic alterations after SCI and that these alterations are primarily driven by time after lesion, irrespective of injury severity. Importantly, SCI induces persistent expression of astrocytic markers in microglia, starting as early as 72 h after injury and maintained up to 6 weeks post-lesion (Figure 7). Microglia reaction after SCI also involves activation of DNA damage pathway in particular pointing toward *Brca1* in both rodents and non-human primates. These data, through the first transcriptomic analysis of microglia at multiple time-points after different lesion severity, provide novel insight into neuroinflammation following CNS lesion.

### Microglia compose the majority of selected eGFP^high^ expressing cells

CX3CR1^+/eGFP^ transgenic mice express eGFP in CNS resident microglia and infiltrating monocyte-derived macrophages. The breakdown of blood spinal cord barrier induced by SCI permits the infiltration of blood monocytes, thus activated macrophages result from a mixed population of resident microglia and infiltrating blood-derived macrophages (Popovich et al., 1996). Therefore, we cannot exclude that our selected cells for RNA-Seq are contaminated with infiltrating monocytes.

Following SCI, an increase in CX3CR1 expression is observed from 72 h post-injury and is further amplified by 1 and 2 weeks (Donnelly et al., 2011). As all CNS macrophages (microglia and monocyte-derived macrophages) express CD11b, they are often discriminated based on the level of CD45 expression (recruited monocytes and activated microglia are CD45^high^ and resident microglia are CD45^low^) (Donnelly et al., 2011). In our study, at 72 h post-injury, it was possible to discriminate two populations of cells (CD45^high^ and CD45^low^), eGFP+ selected cells correspond to both populations, however, infiltrating monocyte-derived macrophages were not detected in the spinal cord lesion before 3 days post-injury (Popovich and Hickey, 2001). Thus, the level of contaminating macrophages is most likely very low.

In contrast, at 1 and 2 weeks post-injury this discrimination was not possible, however, eGFP+ selected cells for RNA-Seq had a CX3CR1^high^Ly6C^neg/low^ antigenic phenotype. Circulating monocytes include two principal subpopulations that both express CX3CR1, the so-called inflammatory monocytes (CX3CR1^low^Ly6C^high^) and a patrolling subgroup (CX3CR1^high^Ly6C^neg/low^ cells) (Geissmann et al., 2003). Using CX3CR1^+/eGFP^ mice to identify monocyte-derived macrophages in a model of contusion SCI through adoptive cell transfer and bone marrow chimera Blomster et al. identified that two subsets of monocytes (CD11b^+^/LY6C^high^/GFP^low^ and CD11b^+^/LY6C^neg/low^/GFP^high^ cells) were recruited in the injured spinal cord 1 week after injury (Blomster et al., 2013). Both subsets were observed around the lesion epicenter and CD11b^+^/LY6C^neg/low^/GFP^high^ macrophages were recruited with lower efficiency, indicating a preferential recruitment of inflammatory monocytes. This preferential concentration of infiltrating monocytes within the margins of the lesion had also been reported earlier using immunohistochemical analysis (Shechter et al., 2009).

Among the CD45^+^CD11b^+^ macrophages, the infiltrating Ly6C^−^Ly6G^+^ (Ly6C^+^, monocytic and Ly6G^+^, granulocytic subset) and Ly6C^+^Ly6G^−^ fractions similarly peaked at 12 h after SCI and returned to the non-injured level at 7 days, while Ly6C^−^Ly6G^−^ fraction increased gradually from 4 h to 7 days, however with a relatively low number of cell (Saiwai et al., 2013). The initial phase following SCI is associated with LY6C^high^CX3CR1^low^ macrophage infiltration corresponding to the “classically activated” cells, while the second phase is characterized by infiltration of LY6C^low^CX3CR1^high^, “alternatively activated” macrophages, which are anti-inflammatory. At 7 days post-injury, LY6C^low^CX3CR1^high^ indeed express anti-inflammatory cytokines (Shechter et al., 2013).

Taken together, since we have selected eGFP^+^ cells located in a 1 cm segment centered on the lesion site and that these cells were CD11^+^/LY6C^neg/low^/GFP^high^ we cannot exclude a low level of contamination with anti-inflammatory macrophages.

### Time-dependent transcriptomic responses in microglia after SCI

We report that responses of microglia after injury are time-dependent and primarily involve proliferation early after injury followed by neuroinflammation at more chronic stages after both HS and FT SCI. Although increased proliferation early after SCI might also be attributed to infiltrating monocyte-derived macrophages, a recent study has shown that CNS resident microglia can proliferate in the absence of peripheral monocytes (Elmore et al., 2014). Moreover, Greenhalgh and David (2014) reported greater proliferation of resident microglia compared to infiltrating monocyte-derived macrophages after SCI (Greenhalgh and David, 2014). Early microglial proliferation has been also reported in mouse models of Alzheimer's disease (Rodriguez et al., 2010) and multiple sclerosis (Ponomarev et al., 2005), suggesting that proliferation of microglia precedes their activation in multiple neuropathological conditions.

To our knowledge, microglia-specific transcriptomic analysis following SCI has not been reported. We recently demonstrated using RNA-Seq analysis of astrocytes at different stages after SCI that, in contrast to microglia, the astrocytic response is both time- and severity-dependent (Noristani et al., 2016). We also establish that SCI induces an astroglial conversion toward neuronal lineage with over 10% of astrocytes expressing classical neuronal progenitor markers with typical immature neuronal morphology (Noristani and Perrin, 2016; Noristani et al., 2016). Together, these findings suggest that glial cells undergo specific transcriptomic alterations after SCI with the microglial response predominantly influenced by the time post-lesion, and the astrocytic response influenced by both time and injury severity.

A prior flow cytometric analysis of microglia/macrophages following SCI revealed an up-regulation of neurotoxic factors (Kroner et al., 2014). However, this study probed only a small number of selected transcripts. Gene profile analysis using microarrays of the whole spinal cord have provided more global insights into SCI-induced gene changes; for example, Kirgel et al. described a predominant over-expression of neurotoxic genes following SCI (Kigerl et al., 2009). A recent RNA-Seq study characterized temporal genome-wide gene expression profiles in the whole rat spinal cord following contusive SCI (Shi et al., 2017). Most de-regulated pathways included immune response, MHC protein complex, antigen processing and presentation cytokine and/or chemokine activity. Spinal cord gene profiling following contusion SCI in non-human primate revealed that the inflammatory response is extended and the onset of glial scar formation is delayed when compared to rodents (Nishimura et al., 2014). Based on transcriptomic profiles the authors discriminated two distinct phases, an acute phase (0–2 weeks post-SCI) characterized by considerable gene expressions changes and a later “steady state” phase. Pathways that are de-regulated in the acute phase include immune responses and production of cytokines and reactive oxygen species (ROS). Using IBA1 immunohistochemistry, a marked infiltration of proliferative microglia/macrophages was reported at the lesion site 1 week after injury in common marmosets (*Callithrix jacchus*) that decreased at 2 weeks post-SCI and disappeared by 6 weeks.

In our study, microglia displayed a dual phenotype with an acute increase in anti-inflammatory factors followed by later upregulation of pro- and anti-inflammatory factors after both HS and FT SCI. These findings suggest a time-dependent shift induced by SCI in microglial phenotype toward a more pro-inflammatory function, as described in a mouse model of ALS (Gerber et al., 2012; Liao et al., 2012). In contrast, others had reported predominant pro-inflammatory activation of microglia after contusion SCI, which may be due to their focus on a relatively small number of selected transcripts rather than genome-wide analyses (Kigerl et al., 2009; Kroner et al., 2014). In addition, contusion injury causes greater inflammatory response, which may induce different effects on microglia activation.

So far, osteopontin (*SPP1*) is the only microglia-derived neurotrophic factor whose positive effect has been reported after SCI (Hashimoto et al., 2007). Here, we demonstrate significant increases in microglia-derived insulin-like growth factor 1 (*Igf1*), arginase 1 (*Arg1*) and progranulin (*Grn*) transcripts, among others, which possess neurotrophic effects both *in vitro* and *in vivo* (Deng et al., 2009; Ryan et al., 2009; Laird et al., 2010). Similarly, so far only 3 of microglia-derived pro-inflammatory factors have been investigated after SCI; namely interleukin-1β (Liu et al., 2008), matrix metalloproteinases 12 (*Mmp12*) (Wells et al., 2003) and NADPH oxidase 2 (*Cybb*) (Cooney et al., 2014). Here we report over-expression of additional microglia-derived proinflammatory transcripts involved in immune/inflammatory response including lysozyme 1 and 2 (*Lyz1* and *Lyz2*), chemokine (C-X-C motif) ligand 9 and 10 (*Cxcl9* and *Cxcl10*), interferon-induced transmembrane protein 1, 2 and 3 (*Ifitm1, Ifitm2*, and *Ifitm3*) after injury, some of which have been shown to inhibit neurite outgrowth *in vitro* and *in vivo* (Ibi et al., 2013). Thus, our results highlight new target genes and raise the possibility that modifying the expression of these transcripts in microglia may reveal their precise contribution in SCI pathophysiology.

An on-going challenge in studying SCI-induced neuroinflammation is the difficulty to discriminate between CNS resident microglia and infiltrating peripheral macrophages, even if recent findings have proposed specific markers to distinguish between these cell types (Chiu et al., 2013; Butovsky et al., 2014; Gosselin et al., 2014; Bennett et al., 2016). Using these markers, Butovsky and colleagues reported clear distinctions in the transcriptomic profile of microglia and macrophages (Butovsky et al., 2014). Furthermore, a recent report suggests that Arg1 is exclusively expressed in infiltrating macrophages, but not microglia, after SCI (Greenhalgh et al., 2016). We have used CX3CR1^+/eGFP^ mice that express eGFP in microglia, monocytes as well as in a subsets of natural killer cells and dendritic cells (Jung et al., 2000). However, microglia display enlarged cell morphology and higher eGFP expression compared to peripheral infiltrating macrophages and we were able to use these properties to preferentially isolate microglia after SCI using flow cytometry selection (Figure S2).

### SCI induces an autologous expression of astrocytic markers in microglia

We also demonstrate injury-induced expression of classical astrocyte-specific markers in a sub-population of microglia. Astroglial markers in microglia were localized within 500 μm of the lesion epicenter, where the highest density of inflammatory cells is found. *In vitro* studies support the ability of microglia to differentiate into GFAP-positive cells through bone morphogenetic protein (BMP) and *Sox2* signaling pathways (Niidome et al., 2008; Nonaka et al., 2009). However, we found no changes in microglia expression of *Sox2* and *BMP* transcripts after injury, suggesting that other signaling pathways may be involved in SCI-induced microglia expression of astrocytic markers *in vivo*. Increased expression of microglial *Gfap, Vim* and *Serpina3n* transcripts was also reported in a mouse model of ALS (Chiu et al., 2013), whilst *in vivo* microglial transformation into astrocyte-like cells were found in a rat model of the disease (Trias et al., 2013). Co-expression of microglia and astrocytic markers has been also found in neoplastic glioblastoma multiform cells associated with increased inflammation (Huysentruyt et al., 2011). Other studies had also shown vimentin expression in reative microglia following injury (Graeber et al., 1988; Wohl et al., 2011). Furthermore, gene expression profiling of primary lymphomas of the CNS (PCNSL) demonstrated that macrophages, activated microglia, and reactive astrocytes within PCNSL samples were SERPINA3-positive (Montesinos-Rongen et al., 2008). Altogether, these findings suggest that pronounced inflammation triggers microglial expression of astrocytic markers. However, it is important to note that SCI-induced astrocytic transcript and protein expression in microglia is restricted to markers of astrogliosis, such as *Gfap, Vim* and *Serpina3n*. Other astrocytic markers necessary for physiological functions of astrocytes including glutamine synthetase (*Glul*), *S100b* and glutamate transporters (*Slc1a1, Slc1a3*), were not altered in microglia after SCI.

The potential role of SCI-induction of astrocytic markers in microglia is currently unknown, although it may suppress the pro-inflammatory pathway. In support of this possibility, hemisected CX3CR1^+/eGFP^ mice that showed a greater SCI-induced increase in astrocytic markers in microglia also displayed reduced microgliosis compared to Aldh1l1-EGFP mouse strain (Noristani et al. unpublished observations). Further studies using cell-specific transcriptomic analysis of microglia that express SCI-induced astrocytic markers are required to uncover their precise involvement in SCI pathophysiology. Alternatively, forced expression of astrocytic markers in microglia could also be used in future studies to investigate the functional role of upregulation of these proteins following SCI.

### Brca1 over-expression in microglia after SCI

We identified the involvement of DNA damage pathway and in particular concomitant changes in the expression level of *Cdk1* (cyclin-dependent kinase 1) and *Brca1* in microglia following SCI. *Cdk1* phosphorylates BRCA1, which reduces BRCA1 activity and function (Johnson et al., 2011). These findings are in line with our previous study in which we reported increased *Brca1* transcript expression in microglia of hSOD1^G93A^ mice, an ALS mouse model, compared to wild type control microglia (Noristani et al., 2015b). Furthermore, *in silico* comparison with data from Chiu et al. (2013) confirmed an age-dependent increase in *Brca1* expression in microglia in a mouse model of ALS (Chiu et al., 2013). Further support for microglia *Brca1* involvement after SCI was found through the dysregulation of over 65% genes linked to *Brca1* and belonging to the DNA damage pathway. Using TUNEL assay to reveal DNA fragmentation in apoptotic cells, Greenhalgh and David (2014) had shown that infiltrating macrophages are more susceptible to apoptosis than CNS resident microglia after SCI (Greenhalgh and David, 2014). Such increased vulnerability to apoptosis is partially due to their reduced proliferation, which in turn increases phagocytic load and intracellular stress in macrophages compared to resident microglia (Greenhalgh and David, 2014).

Here we have shown BRCA1 expression at protein level in *Microcebus murinus*. These data further support our previous study on BRCA1 as a novel microglial marker in human spinal cord (Noristani et al., 2015b). SCI-induced increase in BRCA1 expression in *Microcebus murinus* is also in agreement with our earlier study in which we reported increased BRCA1 expression in ALS spinal cords (Noristani et al., 2015b). BRCA1 is involved in multiple functions including transcription regulation, cell cycle progression, DNA repair (Mantha et al., 2014) and protection against oxidative stress (Bae et al., 2004; Vurusaner et al., 2012). In particular, ROS (reactive oxygen species) play important roles in both pro- and anti-inflammatory states of microglia (David and Kroner, 2011). The functional role of BRCA1 in microglia after SCI remains to be determined, however, one hypothesis may represent an antioxidative defense mechanism.

*Brca1* knockout mice are embryonically lethal (Liu et al., 2014) whereas transgenic mice with reduced *Brca1* expression specifically in neural progenitor cells display increased apoptosis and only survive untill postnatal day 19 (Pao et al., 2014). BRCA1 has also been implicated in the absence of neural tube closure in spina bifida meningomyelocele (Gowen et al., 1996; King et al., 2007).

## Conclusion

In conclusion, our data highlight that responses of microglia following injury are time-dependent with a sequential proliferation and over-expression of anti-inflammatory factors immediately following injury, followed by concomitant upregulation of both pro- and anti-inflammatory factors, irrespective of lesion severity. In addition, we demonstrate injury-induced expression of classical astrocytic markers in microglia starting as early as 72 h post-lesion and continuing up to 6 weeks, after both moderate and severe SCI. Furthermore, we identify the potential involvement of DNA damage pathway and specifically *Brca1* in microglia following SCI. We finally confirm BRCA1 expression in microglia that is significantly increased in a non-human primate model of SCI. The up-regulation of *Brca1* in SCI-microglia could be anecdotal, but we already demonstrated using a similar transcriptomic approach that BRCA1 is up-regulated in human microglia of ALS patient (Noristani et al., 2015b). In summary, our data represent the first microglia transcriptomic analyses at multiple time-points after different lesion severity and provide new insight into their response after SCI.

## Author contributions

HN participated in the design of the study, performed majority of the experiments, analyzed the data and contributed to the writing of the manuscript; YG participated in mouse SCI, flow cytometry and Brca1 analysis in *Microcebus murinus;* JS participated in mouse SCI and flow cytometry; NL participated in *Microcebus murinus* SCI; ML, participated in *Microcebus murinus* SCI and data analysis; NM, participated in *Microcebus murinus* management; HH participated in mouse colony management, flow cytometry and data analysis; FP conceptualized the research, designed the project, participated in the analysis and data interpretation, drafting the work and final approval.

## Funding

This work was supported by the Spanish Government, Plan Nacional de I+D+I 2008-2011 and ISCIII- Subdirección General de Evaluación y Fomento de la investigación (PI10/00709) [to FP] and the Government of the Basque Country grant (Proyectos de Investigacion Sanitaria and Fondo Comun de Cooperacion Aquitania-Euskadi) [to FP], the French Government, ANR-FNS grant, GliALS (N° ANR-14-CE36-0009-01) [to FP], the patient organizations “Demain Debout Aquitaine” [to YG and HN] and “Verticale” [to FP and HN].

### Conflict of interest statement

The authors declare that the research was conducted in the absence of any commercial or financial relationships that could be construed as a potential conflict of interest.

## Supplementary material

Figure S1 **Specific microglial eGFP expression in CX3CR1^+/eGFP^ mice spinal cord**. Schematic drawing of longitudinal spinal cords from either non-injured control or following FT. The red square illustrates the lesion site and reference frames display on the field of views. Confocal micrographs showing microglial eGFP expression in non-injured CX3CR1^+/eGFP^ mice spinal cord using IBA1 immunostaining **(A–C)**. Fluorescent images showing microglial eGFP expression in non-injured **(D–F)** and injured (FT) spinal cord **(G–I)** of CX3CR1^+/eGFP^ mice using S100-beta immunostaining. Scale bars **(A**, **D–I)**: 50 μm, **(B,C)**: 10 μm.

Figure S2 **Flow cytometry analysis**. Representative flow cytometry analysis dot plots displaying control **(A)** and eGFP^high^-expressing microglia profiles from non-injured **(B)** as well as after HS **(C)** and FT SCI **(D)**. Surrounded areas, designed as “P4” represent sorted cells that correspond to the eGFP^high^-expressing cells further analyzed using RNAseq. The X- and Y-axis display fluorescent intensity and cell size, respectively. Representative flow cytometry analysis dot plots of cells in non-injured samples **(E)**, as well as at 72 h **(F)**, 1 week **(G)**, and 2 weeks **(H)** after HS according to CD11b and CD45 expression. Cells were first gated based on size and granularity with small size events being gated-out (not shown). Definition of the different cell populations recovered after sucrose gradient according to their expression of LY6C and CD45 in non-injured samples **(I)**, as well as at 72 h **(J)**, 1 week **(K)**, and 2 weeks **(L)**. Cells were first gated based on size and granularity with small size events being gated-out (not shown). Cell populations were then separated in CD45^−^/LY6C^−^, CD45^−^/LY6C^+^, CD45^+^/LY6C^−^, and CD45^+^/LY6C^+^ expressing cells. Characterization of eGFP expression levels in the different cell populations recovered after sucrose gradient (as defined in **S2I–L**). Only the CD45^+^/LY6C^−^ population exhibit high eGFP expression level. All CD45^+^/LY6C^+^ cells are GFP^−^ or GFP^low^ expressing cells **(M–P)**. All other cell populations are GFP^−^. Vertical bars indicate the lower threshold for eGFP expression in sorted cells (see **S2A–D**). Representative flow cytometry analysis dot plots displaying eGFP^high^ expressing cells in non-injured samples **(Q)**, as well as 72 h **(R)**, 1 week **(S)**, and 2 weeks **(T)** after HS. Characterization of LY6C expression levels in the GFP^high^-sorted cells. Whatever the experimental conditions, GFP^high^-sorted cells are Ly6C^−^ **(U)**. Expression of LY6C^+^ in CD45^+^/LY6C^+^ cell population (as gated in S2I) is shown as control.

Figure S3 **Specific microgial transcript over-expression after SCI in CX3CR1^+/eGFP^ mice**. Bar graphs displaying specific over-expression of microglia-specific transcripts at different stages after HS **(A)**. Values are actual fold change. Bar graphs indicating up-regulation of *Serpina3n* transcript expressions in microglia at different time-point after HS and FT SCI **(B,C)**. Values are actual fold change, t-test between HS or FT as compared to non-injured at a given time point (^**^*p* < 0.01; ^***^*p* < 0.001).

Figure S4 **Microglia responses after SCI are time-dependent irrespective of lesion severity**. Schematic diagram displaying the multiple comparisons carried out to analyze deregulated genes in microglia at multiple time-points after HS and FT SCI **(A)**. Table illustrating the number of deregulated transcripts in each comparisons **(B)**. Note that no deregulated genes were found when directly comparing HS and FT SCI at 72 h post-lesion, whereas only 8 and 1 deregulated transcripts were found after direct comparison between HS and FT SCI at 1 and 2 weeks, respectively. Relationship between average expression and log 2 of the fold-change in microglia after direct comparisons between HS and FT groups at 72 **(C)**, 1 week **(D)**, and 2 weeks **(E)**. Horizontal blue lines indicate the cut off criterion used to define differentially expressed genes with a FC >2 (logFC comprised between −1 and 1) and a significant false discovery rate (FDR) values (*p* < 0.05). Red points indicate significantly deregulated genes following injury.

Figure S5 **Induction of neural development pathways in microglia after SCI**. Gene ontology pathway map analysis displaying the induction of neural development pathway in microglia after SCI. Thermometers indicate deregulated genes (red: up-regulated; blue: down-regulated). Interactions between objects: green (positive or activation); red (negative or inhibition); grey (unspecified); B: Binding (physical interaction between molecules);  Binding protein,  Generic enzyme.

Figure S6 **Increased IBA1 reactivity 3 months after SCI in *Microcebus murinus***. Bright field micrographs displaying IBA1-positive microglia rostral **(A–C)**, within **(D–F)** and caudal **(G–I)** to the lesion site at 3 months following spinal cord hemisection in *Microcebus murinus*. Note similar to BRCA1 immunostaining adjacent sections stained with IBA1 displayed identical lablelling and microglial morphology with long/thin processes caudal to the lesion sites **(I)**, and activated/amoeboid morphology with enlarged cell bodies with short/thick processes that were mainly evident within the dorsal funiculus rostral to the lesion site **(C)** and adjacent to the lesion epicentre **(I)**. Scale bar **(A,D,G)**: 500 μm, **(B,E,H)**: 200 μm, **(C,F,I)**: 50 μm.

Table S1 **Database of differential expression comparison of activated microglia RNA-Seq data relative to non-injured control microglia at 72 h, 1 and 2 weeks after hemisection and full transection injuries**.

Table S2 **Pathway analysis of differentially expressed genes in activated microglia after hemisection and full transection injuries**.

Table S3 **Database of the expression level of cellular markers (microglia, neuronal, astrocyte and oligodendrocyte)**. Differentially expressed genes amongst these cellular markers, data relative to non-injured control microglia at 72 h, 1 and 2 weeks after hemisection and full transection injuries. FC, Fold change; FDR, false discovery rate.

## Abbreviations

ALS: amyotrophic lateral sclerosis
BMP: bone morphogenetic proteins
Brca1: breast cancer susceptibility gene 1
CNS: central nervous system
CPM: counts per million
eGFP: enhanced green fluorescent protein
FACS: fluorescence-activated cell sorting
FC: fold-change
FT: full transection
GFAP: glial fibrillary acidic protein
HS: hemisection
MMRRC: Mutant Mouse Regional Resource Centre
NI: non-injured
PBS: phosphate base saline
PFA: paraformaldehyde
RNA-Seq: RNA sequencing
SCI: spinal cord injury
SEM: standard error of the mean.
