# Supplementary figures and images for: RNA-Seq Analysis of Microglia Reveals Time-Dependent Activation of Specific Genetic Programs following Spinal Cord Injury
Source: Front Mol Neurosci. 2017 Apr 3;10:90. doi: 10.3389/fnmol.2017.00090 (PMC5376598; doi:10.3389/fnmol.2017.00090)

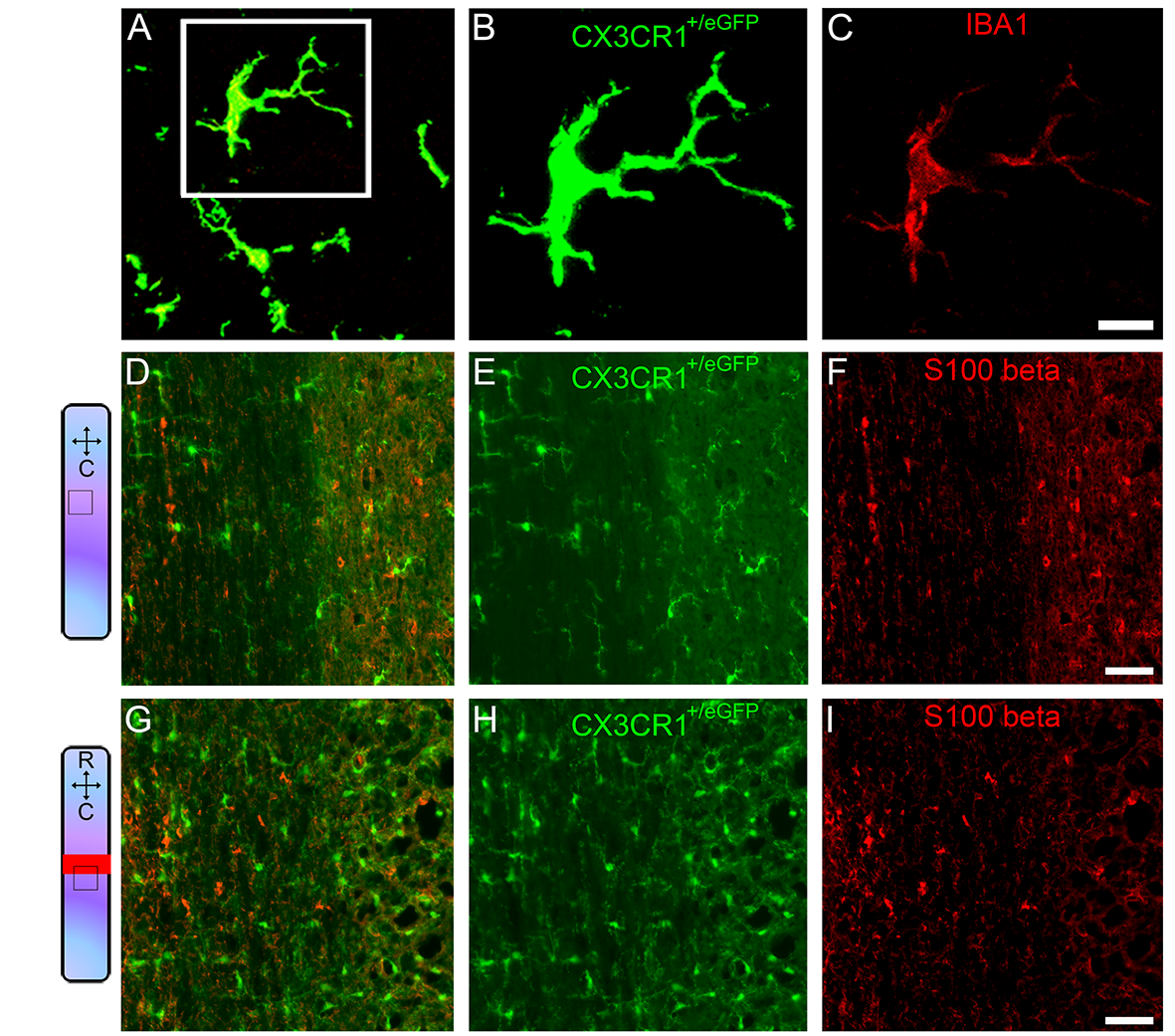

Supplement: Figure S1 — Specific microglial eGFP expression in CX3CR1+/eGFP mice spinal cord. Schematic drawing of longitudinal spinal cords from either non-injured control or following FT. The red square illustrates the lesion site and reference frames display on the field of views. Confocal micrographs showing microglial eGFP expression in non-injured CX3CR1+/eGFP mice spinal cord using IBA1 immunostaining (A–C). Fluorescent images showing microglial eGFP expression in non-injured (D–F) and injured (FT) spinal cord (G–I) of CX3CR1+/eGFP mice using S100-beta immunostaining. Scale bars (A, D–I): 50 μm, (B,C): 10 μm. [file Image1.TIF]

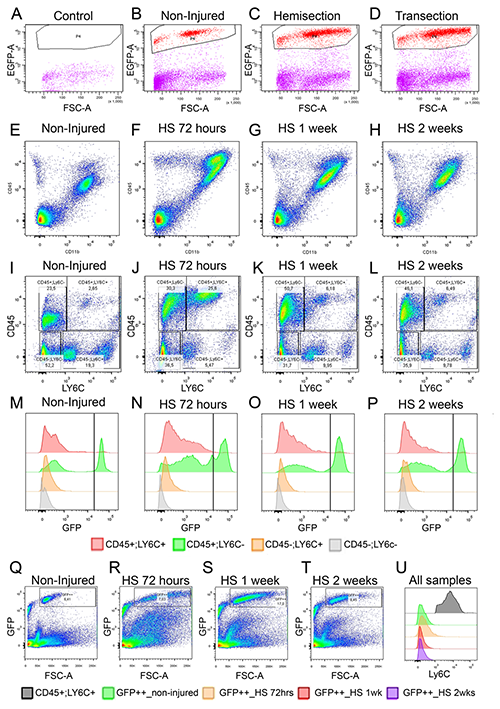

Supplement: Figure S2 — Flow cytometry analysis. Representative flow cytometry analysis dot plots displaying control (A) and eGFPhigh-expressing microglia profiles from non-injured (B) as well as after HS (C) and FT SCI (D). Surrounded areas, designed as “P4” represent sorted cells that correspond to the eGFPhigh-expressing cells further analyzed using RNAseq. The X- and Y-axis display fluorescent intensity and cell size, respectively. Representative flow cytometry analysis dot plots of cells in non-injured samples (E), as well as at 72 h (F), 1 week (G), and 2 weeks (H) after HS according to CD11b and CD45 expression. Cells were first gated based on size and granularity with small size events being gated-out (not shown). Definition of the different cell populations recovered after sucrose gradient according to their expression of LY6C and CD45 in non-injured samples (I), as well as at 72 h (J), 1 week (K), and 2 weeks (L). Cells were first gated based on size and granularity with small size events being gated-out (not shown). Cell populations were then separated in CD45−/LY6C−, CD45−/LY6C+, CD45+/LY6C−, and CD45+/LY6C+ expressing cells. Characterization of eGFP expression levels in the different cell populations recovered after sucrose gradient (as defined in S2I–L). Only the CD45+/LY6C− population exhibit high eGFP expression level. All CD45+/LY6C+ cells are GFP− or GFPlow expressing cells (M–P). All other cell populations are GFP−. Vertical bars indicate the lower threshold for eGFP expression in sorted cells (see S2A–D). Representative flow cytometry analysis dot plots displaying eGFPhigh expressing cells in non-injured samples (Q), as well as 72 h (R), 1 week (S), and 2 weeks (T) after HS. Characterization of LY6C expression levels in the GFPhigh-sorted cells. Whatever the experimental conditions, GFPhigh-sorted cells are Ly6C− (U). Expression of LY6C+ in CD45+/LY6C+ cell population (as gated in S2I) is shown as control. [file Image2.tif]

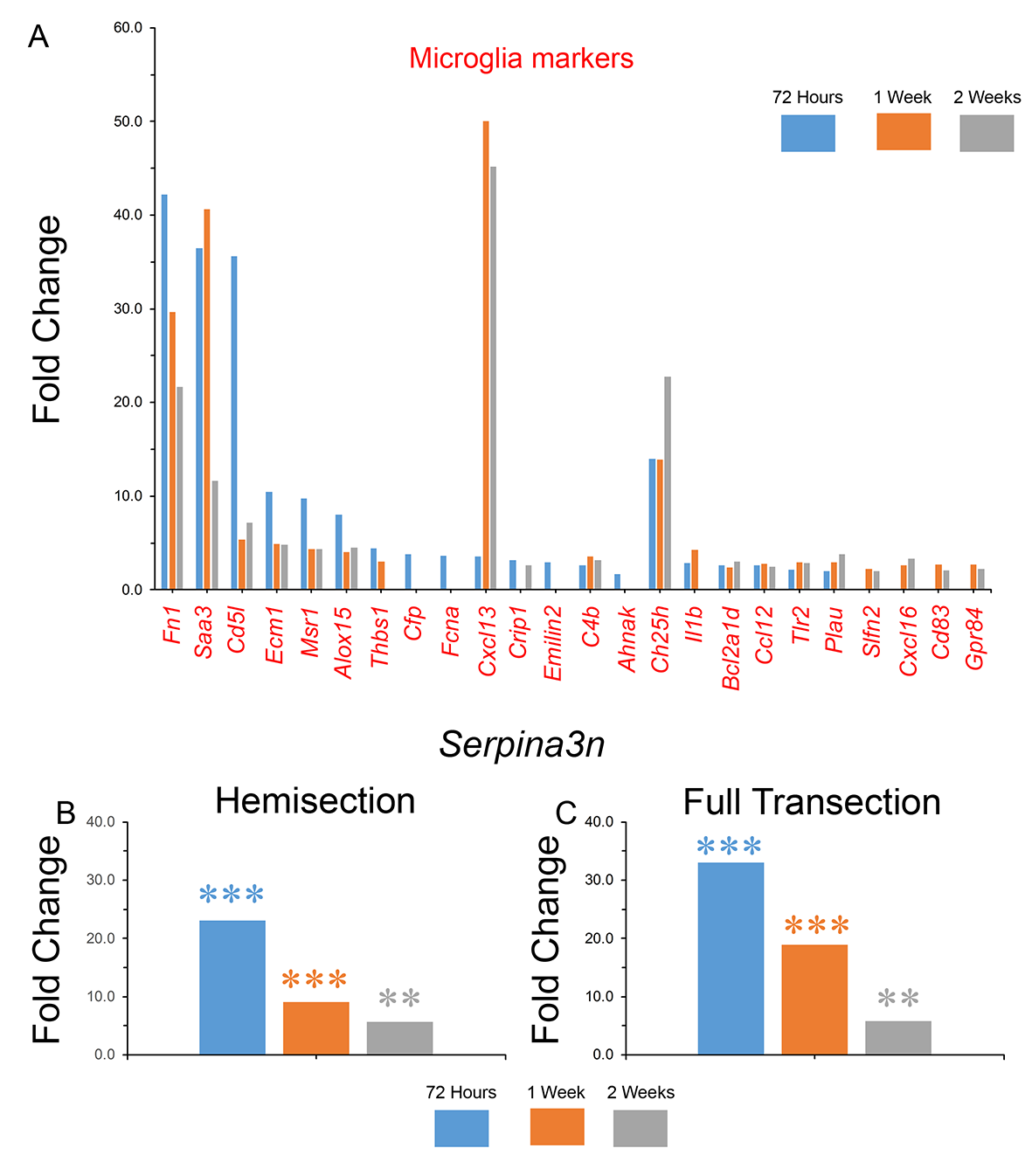

Supplement: Figure S3 — Specific microgial transcript over-expression after SCI in CX3CR1+/eGFP mice. Bar graphs displaying specific over-expression of microglia-specific transcripts at different stages after HS (A). Values are actual fold change. Bar graphs indicating up-regulation of Serpina3n transcript expressions in microglia at different time-point after HS and FT SCI (B,C). Values are actual fold change, t-test between HS or FT as compared to non-injured at a given time point (**p < 0.01; ***p < 0.001). [file Image3.TIF]

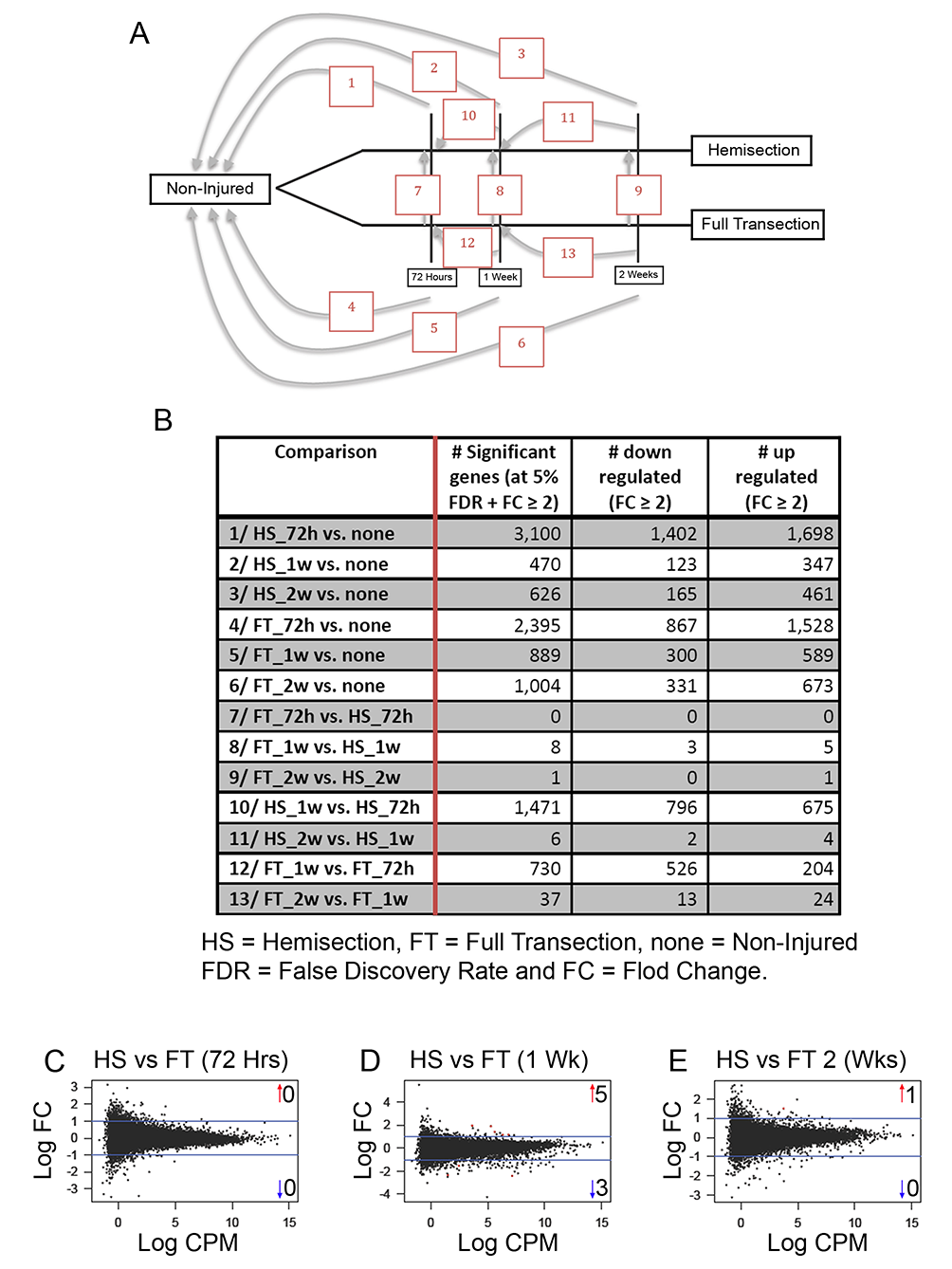

Supplement: Figure S4 — Microglia responses after SCI are time-dependent irrespective of lesion severity. Schematic diagram displaying the multiple comparisons carried out to analyze deregulated genes in microglia at multiple time-points after HS and FT SCI (A). Table illustrating the number of deregulated transcripts in each comparisons (B). Note that no deregulated genes were found when directly comparing HS and FT SCI at 72 h post-lesion, whereas only 8 and 1 deregulated transcripts were found after direct comparison between HS and FT SCI at 1 and 2 weeks, respectively. Relationship between average expression and log 2 of the fold-change in microglia after direct comparisons between HS and FT groups at 72 (C), 1 week (D), and 2 weeks (E). Horizontal blue lines indicate the cut off criterion used to define differentially expressed genes with a FC >2 (logFC comprised between −1 and 1) and a significant false discovery rate (FDR) values (p < 0.05). Red points indicate significantly deregulated genes following injury. [file Image4.TIF]

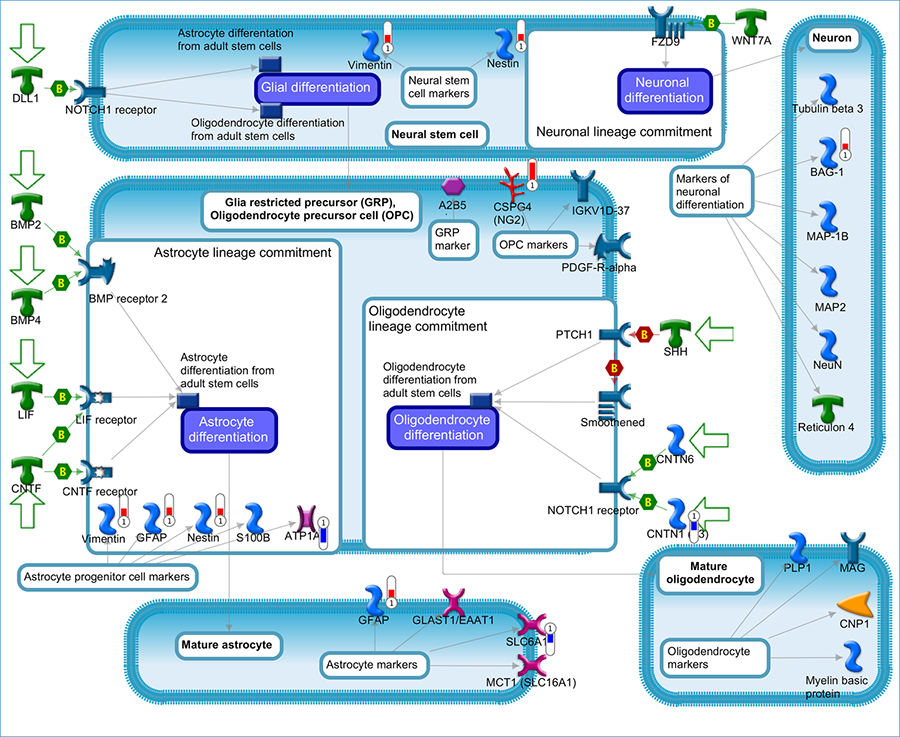

Supplement: Figure S5 — Induction of neural development pathways in microglia after SCI. Gene ontology pathway map analysis displaying the induction of neural development pathway in microglia after SCI. Thermometers indicate deregulated genes (red: up-regulated; blue: down-regulated). Interactions between objects: green (positive or activation); red (negative or inhibition); grey (unspecified); B: Binding (physical interaction between molecules); Binding protein, Generic enzyme. [file Image5.tif]

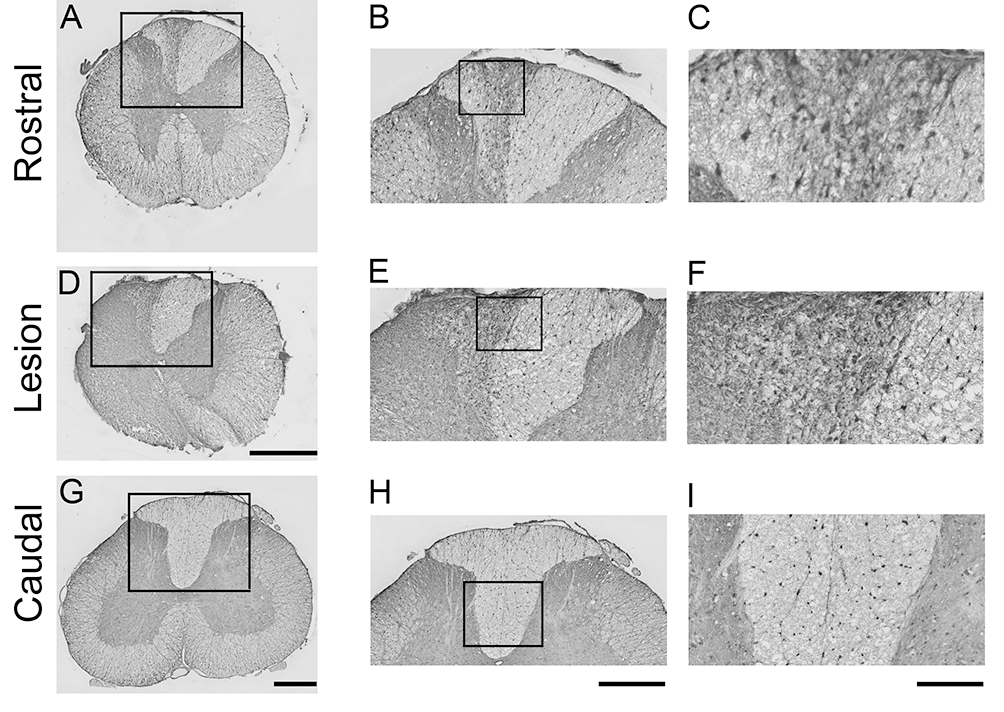

Supplement: Figure S6 — Increased IBA1 reactivity 3 months after SCI in Microcebus murinus. Bright field micrographs displaying IBA1-positive microglia rostral (A–C), within (D–F) and caudal (G–I) to the lesion site at 3 months following spinal cord hemisection in Microcebus murinus. Note similar to BRCA1 immunostaining adjacent sections stained with IBA1 displayed identical lablelling and microglial morphology with long/thin processes caudal to the lesion sites (I), and activated/amoeboid morphology with enlarged cell bodies with short/thick processes that were mainly evident within the dorsal funiculus rostral to the lesion site (C) and adjacent to the lesion epicentre (I). Scale bar (A,D,G): 500 μm, (B,E,H): 200 μm, (C,F,I): 50 μm. [file Image6.TIF]
